# Association Between Serum Galectin-3 Levels and Coronary Stenosis Severity in Patients With Coronary Artery Disease

**DOI:** 10.3389/fcvm.2022.818162

**Published:** 2022-02-07

**Authors:** Mingxing Li, Kai Guo, Xuansheng Huang, Li Feng, Yong Yuan, Jiewen Li, Yi Lao, Zhigang Guo

**Affiliations:** ^1^Division of Cardiology, Huiqiao Medical Centre, Nanfang Hospital, Southern Medical University, Guangzhou, China; ^2^Department of Cardiology, Zhongshan People's Hospital, Zhongshan, China

**Keywords:** coronary artery disease, galectin-3, syntax score, prognosis, acute coronary syndrome

## Abstract

**Background:**

The relationship between galectin-3 (Gal-3) and coronary artery disease (CAD) has not been fully elucidated.

**Aim:**

This study aimed to determine the relationship between the presence and severity of CAD and serum Gal-3 levels.

**Patients and Methods:**

Three-hundred thirty-one consecutive CAD patients were enrolled as the study group. An additional 62 patients without CAD were enrolled as the control group. Serum Gal-3 levels were separately compared between the non-CAD and CAD groups, among the stable CAD and Acute coronary syndrome (ACS) groups, and between CAD patients with low and high SYNTAX scores (SSs). The 1-year cumulative rate of major adverse cardiac events (MACEs) was also compared among ACS patients by Gal-3 levels.

**Results:**

Serum Gal-3 was significantly higher in the CAD group than in the non-CAD group 3.89 (0.16–63.67) vs. 2.07 (0.23–9.38) ng/ml, *P* < 0.001. Furthermore, serum Gal-3 was significantly higher in the non-ST-segment elevation ACS (NSTE-ACS) group than that in the stable CAD group, 4.72 (1.0–16.14) vs. 2.23 (0.65–23.8) ng/ml, *P* = 0.04 and higher in the ST-segment elevation myocardial infarction (STEMI) group than that in the stable CAD group 7.87 (0.59–63.67) vs. 2.23 (0.65–23.8) ng/ml, *P* < 0.001. Serum Gal-3 level was an independent predictor of ACS compared with stable CAD group (OR = 1.131, 95% CI: 1.051–1.217, *P* = 0.001) as well as high SS (OR = 1.030, 95% CI: 1.021–1.047, *P* = 0.038) after adjust other confounding risk factors. Acute coronary syndrome patients with Gal-3 levels above the median (gal-3 = 4.78 ng/ml) showed a higher cumulative MACE rate than those with Gal-3 levels below the median. After adjusting other confounding risk factors, Gal-3 remained an independent risk factor for the cumulative rate of MACEs in ACS patients (6% higher rate of MACEs incidence per 1 ng/ml increment of Gal-3).

**Conclusion:**

Galectin-3 correlated with the presence of CAD as well as coronary stability and complexity. Galectin-3 may be valuable in predicting mid-term prognosis in ACS patients.

## Introduction

Cardiovascular diseases (CVDs) remain the leading cause of death worldwide (1). Atherosclerosis is a major cause of stroke and CVDs (2) and is characterized by the excessive accumulation of lipoprotein in macrophages, monocyte chemo-attraction in vascular lesions, and the infiltration of vascular smooth muscle cells (VSMCs) into the sub-endothelial space (3). Inflammation mediated by macrophages plays an important role in the initiation and progression of atherosclerosis (3, 4).

Galectin-3 (Gal-3) is a pro-inflammatory cytokine that is mainly secreted by activated macrophages (5). It is a circulating 35 kDa β-galactosidase-binding lectin and the unique chimera-like galectin member of the vertebrate family (6). It has one C-terminal carbohydrate recognition domain connected to a long N-terminal domain, and in the human genome, it is encoded solely by LGALS3, located on chromosome 14, locus q21-22 (5, 6). Galectin-3 has several biological functions, including intracellular and short-distance signaling, regulation of gene expression, cell-to-cell interaction, and exchanges between cells and the extracellular matrix (ECM) (7, 8). Over-expression of Gal-3 has been observed in patients with decompensated congestive heart failure (CHF). In addition, Gal-3 may play an important role in the inflammatory response, fibrosis and scar formation, cardiac remodeling, and heart failure in the clinical setting of acute myocardial infarction (AMI) (9).

Recently, clinical data have suggested that Gal-3 is closely correlated with coronary atherosclerosis (9–13). However, the precise role of Gal-3 in coronary artery disease (CAD) has not yet been fully elucidated, and more data are needed to systemically explore the association between serum Gal-3 levels and atherosclerotic plaque burden and stability. We therefore performed a retrospective cohort clinical study to explore the relationship between peripheral Gal-3 levels and the presence of CAD as well as plaque burden and stability. The value of Gal-3 in predicting mid-term prognosis in acute coronary syndrome (ACS) patients was also evaluated.

## Patients and Methods

### Study Population

This is a single-center, retrospective cohort study. From January 1 to December 1, 2018, we continuously enrolled 393 consecutive patients who underwent coronary angiography due to suspected coronary heart disease. Patients who met one of the following criteria were excluded: (1) patients with acute or chronic infectious diseases or autoimmune diseases or recently used drugs that affect the immune response; (2) patients with severe heart failure, liver or kidney dysfunction; (3) patients complicated with any kind of tumor; and (4) patients who refused to sign the informed consent form and did not want to participate in this research.

### Methods

First, the enrolled patients were divided into the control (non-CAD group, *n* = 62) and CAD groups (*n* = 331). Serum Gal-3 levels were compared between the two groups. Then, we did subgroup analysis and all the enrolled CAD patients were further divided into the stable CAD, non-ST-segment elevation ACS (NSTE-ACS) group and ST-segment elevation myocardial infarction (STEMI) group. Serum Gal-3 levels were compared among the three groups. The SYNTAX score (SS) was then calculated for all CAD patients, and the serum Gal-3 level was also compared between the low SS (<22) group and the high SS (≥22) group. We performed multivariate logistic regression analysis to explore the correlation between serum Gal-3 and ACS compared with stable CAD and high SS compared with low SS.

### Clinical Data Collection

The patient's medical history, sex, age, body mass index (BMI), and laboratory test results such as white blood cell (WBC) count, the serum creatinine (CR), fasting glucose (FG) level, glycosylated hemoglobin level, hs-CRP, left atrial diameter, and left ventricular ejection fraction (LVEF) according to echocardiography, Killip grades, medication treatment during hospitalization were obtained and collected from the hospital medical record system.

### Definitions

Coronary artery disease was defined as ≥50% luminal diameter stenosis of at least one major epicardial coronary artery. ST-segment elevation myocardial infarction was defined as follows: (i) There is evidence of myocardial injury which is defined as an elevation of cardiac troponin values with at least one value above the 99th percentile upper reference limit. (ii) Patients with persistent chest discomfort or other symptoms suggestive of ischemia. (iii) ST-segment elevation in at least two contiguous leads (14). Non-ST-segment elevation ACS was defined according to 2020 European Society of Cardiology (ESC) Guidelines (15) and was stated as follows: patients with acute chest discomfort but no persistent ST-segment elevation. ECG changes may include transient ST-segment elevation, persistent or transient ST-segment depression, T-wave inversion, flat T waves, or pseudonormalization of T waves; or the ECG may be normal.

Stable CAD was defined according to the 2013 ESC guidelines recommended (16).

### Galectin-3 Detection

The blood sample was collected from the cubital vein in an ethylene diaminetetraacetic acid (EDTA) vacuum tube, placed in a 4°C refrigerator, allowed to stand for 4 h, and centrifuged at 1,000 × g for 15 min. Then, the supernatant was collected and placed in a −80°C refrigerator for later inspection. The assay was performed and calibrated according to the manufacturer's protocol using an enzyme-linked immunosorbent assay. Measurements were performed in duplicate, and the results were averaged. The standard curve ranged between 0.47 and 30.0 ng/ml. The limit of detection was 0.29 ng/ml, and the intra- and inter-assay reproducibility coefficients of variation were 7.5 and 5.4%, respectively.

### Coronary Angiography and SYNTAX Score

All patients underwent coronary angiography in the catheter lab of the Department of Cardiology in our hospital. Coronary arteriography was conducted using the standard Judkins technique (17). The results of the angiography were judged by two experienced specialists. The SS of each of the selected patients in this study was calculated by the online SS calculator version 2.1 (www.syntaxscore.com).

### Follow-Up

All enrolled ACS patients were followed up for 12 months. Major adverse cardiac events (MACEs) are defined as re-infarction, worsening heart failure, or recurrent angina. The survival time without a MACE is the time before the first MACE during follow-up. Data were obtained through outpatient or telephone interviews.

### Statistical Analysis

Statistical analysis was performed using the SPSS package, version 17.0 (Chicago, Illinois, USA). To test differences between the groups, the Student's *t*-test was used for numerical variables with a regular distribution, and the Mann–Whitney *U*-test was employed if there was an irregular distribution. Categorical variables were analyzed with the chi-squared and Fisher's exact tests. Logistic regressions were used to assess the relationship between serum gal-3 and ACS, high SS. The initial model adjusted for age and gender. A second model additionally adjusted for SBP, WBC, Cr, LDL-c, apoB 100, FG, and LVEF. Kaplan-Meier analysis was used to compare the cumulative rate of MACEs. We used the median Gal-3 level as the cut-off value to divide the ACS patients into a high Gal-3 (>4.78 ng/ml, *n* = 118) and low Gal-3 (≤ 4.78 ng/ml, *n* = 118) group. The 1-year cumulative rate of MACEs was compared between ACS patients whose Gal-3 level was above and below the median level. Multivariate logistic regression analysis was performed to explore the correlation between Gal-3 and 1 year of MACEs in ACS patients in this study. A *p*-value of < 0.05 was regarded as statistically significant.

## Results

### Serum Galectin-3 Expression in Patients With CAD vs. No CAD

The baseline characteristics of the patients in CAD and control groups are shown in Table 1. Compared with that in the no-CAD group, a higher proportion of the patients in the CAD group had a history of hypertension. The biochemical results showed that the WBC count and fasting blood glucose, glycosylated hemoglobin, CR, and hs-CRP levels were higher in the CAD group than in the non-CAD group. The levels of low-density lipoprotein cholesterol (LDL-C) and ApoB100 were also higher in the CAD group. The level of serum Gal-3 in the CAD group was significantly higher than that in the non-CAD group, 3.89 (0.16–63.67) vs. 2.07 (0.23–9.38) ng/ml, *P* < 0.001. To further clarify the relationship, we subsequently adjusted confounding risk factors such as age, gender, SBP, WBC, Cr, LDL-c, apoB 100, FG, and LVEF. Serum Gal-3 remained an independent risk factor for CAD with an increase of 1 ng/ml in Gal-3 associated with a 21% higher rate of presence of CAD (OR = 1.21, 95% CI: 1.07–1.38, *P* = 0.003, Table 2).

**Table 1:** The baseline clinical and biochemical characteristics of the study with CAD or no-CAD.

**Variable**	**no-CAD, *n* = 62**	**CAD, *n* = 331**	***P***
Age, years	60.35 ± 10.36	60.81 ± 11.78	0.601
Gender male sex, % (*n*)	49 (79.0)	268 (80.9)	0.833
BMI, kg/m^2^	24.51 ± 2.22	24.10 ± 2.58	0.248
Hypertension, % (*n*)	26 (41.9)	195 (58.9)	0.013^*^
DM, % (*n*)	8 (12.9)	65 (19.6)	0.285
History of hyperlipidemia, % (*n*)	1 (1.6)	12 (3.6)	0.701
Smoking, % (*n*)	23 (37.1)	136 (41.1)	0.330
Family history of CAD, % (*n*)	0 (0)	9 (2.7)	0.365
Systolic blood pressure, mm Hg	134.87 ± 22.83	142.32 ± 23.33	0.420
WBC, 10^9^/L	7.33 ± 1.74	9.51 ± 4.91	<0.001^*^
Hb, g/L	139.27 ± 15.48	140.31 ± 21.89	0.704
Fasting blood glucose, mmol/L	5.53 ± 1.18	6.55 ± 2.29	<0.001^*^
Hb A1c, %	6.11 ± 1.51	6.35 ± 1.41	0.026^*^
Creatinine, μmol/l	71 (43–131)	85 (33–187)	<0.001^*^
hs-CRP, mg/L	5.16 ± 1.72	8.22 ± 1.13	0.029^*^
Total cholesterol, mmol/L	4.26 ± 0.94	4.52 ± 1.23	0.119
HDL cholesterol, mmol/L	0.68–1.99	0.27–4.62	0.829
LDL cholesterol, mmol/L	2.45 ± 0.74	2.81 ± 1.08	0.028^*^
Triglycerides, mmol/L	1.7 (0.56–5.82)	1.52 (0.49–7.93)	0.176
ApoA1, mg/dl	1.09 ± 0.17	1.11 ± 0.25	0.747
ApoB100, mg/dl	0.84 ± 0.22	1.02 ± 0.39	0.001^*^
Lpa, mg/dl	176 (0–885)	168 (0–3,440)	0.334
LV diameter, mm	45.5 (31–59)	46.5 (28–69)	0.082
LVEF,%	65 (50–75)	59 (22–80)	0.016^*^
Serum Gal-3, ng/ml	2.07 (0.23–9.38)	3.89 (0.16–63.67)	<0.001^*^

*CAD, coronary artery disease; BMI, body mass index; DM, diabetic mellitus; WBC, white blood cell; Hb, hemoglobin; HbA1C, hemoglobin A1C; CRP, C-reactive protein; TC, total cholesterol; HDL-C, high density lipoprotein cholesterol; LDL-C, low-density lipoprotein cholesterol; TG, triglyceride; ApoA1, apolipoprotein A1; ApoB, apolipoprotein B; LP(a), Lipoprotein(a); LVEF, left venticular ejection fraction*.

* *P < 0.05*.

**Table 2:** Association between serum Gal-3 and presence of CAD.

**Gal-3**	**Incident rate**	**Model 1**		**Model 2**		**Model 3**	
		**OR (95%CI)**	***P*-value**	**OR (95%CI)**	***P*-value**	**OR (95%CI)**	***P*-value**
Per ng/ml	331/393	1.30 (1.13, 1.50)	<0.001	1.21 (1.07, 1.37)	0.002	1.21 (1.07, 1.38)	0.003

*Model 1, adjusted for age, gender; Model 2, further adjusted for SBP, WBC, Cr, LDL-c, apoB 100; Model 3, further adjusted for fasting glucose, ejection fraction*.

### Serum Galectin-3 Expression in Patients With STEMI vs. NSTE-ACS vs. Stable CAD

**Figure 1:**
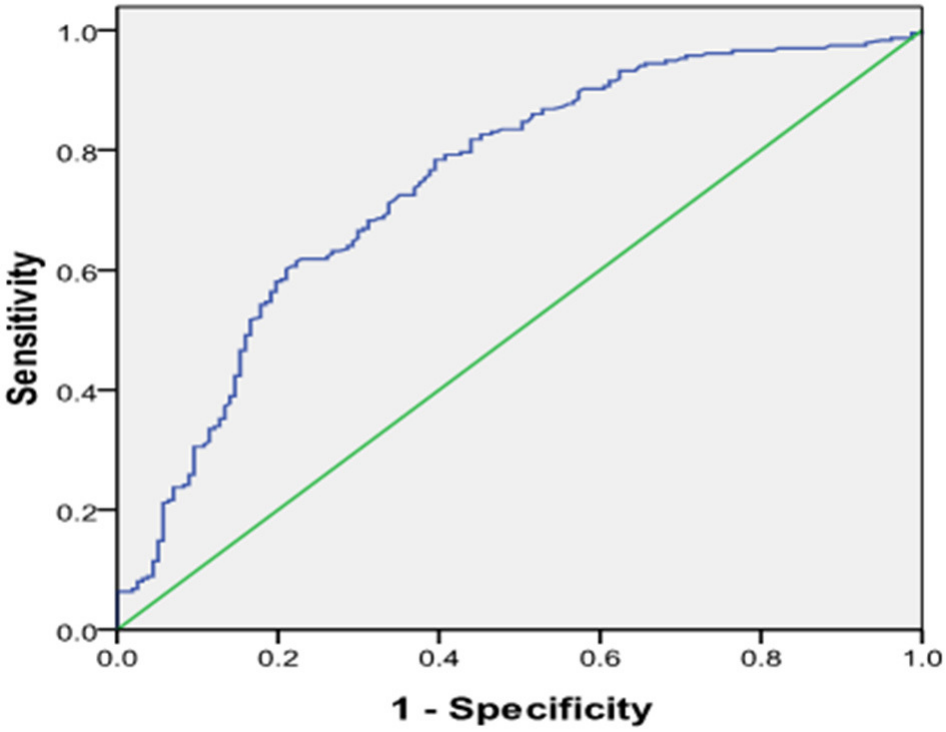
Receiver operating characteristic (ROC) plot of serum level of galetcin-3 predicting presence of ACS. Area under the curve was 0.746, 95% CI (0.696–0.797), *P* < 0.001. When the cut-off value of galectin-3 was 3.93 ng/ml, the sensitivity was 60% with specificity 79% for predicting the presence of ACS.

We further divided the CAD patients into the stable coronary heart disease group (stable-CAD, *n* = 95), Non-ST segment elevation myocardial infarction (NSTE-ACS, *n* = 97) and STEMI (*n* = 139). The serum Gal-3 level in the NSTE-ACS group was higher than those in the stable CAD group, 4.72 (1.0–16.14) vs. 2.23 (0.65–23.8) ng/ml, *P* = 0.04. The same trend was found in the STEMI group compared with the stable-CAD group 7.87 (0.59–63.67) vs. 2.23 (0.65–23.8) ng/ml, *P* < 0.001 see in Table 3. Univariate and multivariate logistic regression analysis showed that after adjusting for other risk factors, Gal-3 was an independent risk factor for ACS, with an OR = 1.131 (95% CI: 1.051–1.217, *P* = 0.001) (Table 4). Receiver operator characteristic (ROC) analysis showed that the area under the curve for serum Gal-3 level predicting ACS was 0.746 (95% CI: 0.696–0.797) and the best cut-off value was 3.93 ng/ml, with a specificity of 79% and a sensitivity of 60% (Figure 1).

**Table 3:** The baseline clinical and biochemical characteristics of STEMI vs NSTE-ACS vs Stable CAD vs. non-CAD in the study.

	**No CAD**	**Stable CAD**	**NSTE-ACS**	**STEMI**	***P-*value**
	***n* = 62**	***n* = 95**	***n* = 97**	***n* = 139**	
Age, years	60.35 ± 10.36	61.00 ± 10.61	61.95 ± 10.17	58.49 ± 13.19	0.058
Gender male sex, % (*n*)	39 (62.9)	57 (60)	82 (84.5)	120 (89.4)	<0.001
BMI, kg/m^2^	24.51 ± 2.22	24.18 ± 2.53	24.17 ± 2.50	24.00 ± 2.67	0.483
Hypertension, % (*n*)	26 (41.9)	59 (62.1)	60 (75.9)	76 (54.6)	0.026
DM, % (*n*)	8 (12.9)	21 (21.1)	22 (22.7)	22 (15.8)	0.292
History of hyperlipidemia, % (*n*)	1 (1.6)	6 (6.3)	3 (3.1)	3 (2.2)	0.096
Smoking, % (*n*)	23 (37.1)	29 (30.5)	37 (38.1)	70 (50.4)	0.014
Family history of CAD, % (*n*)	0 (0)	3 (3.1)	4 (4.1)	2 (1.4)	0.507
Systolic blood pressure, mm Hg	142.32 ± 23.33	137.93 ± 20.50	136.06 ± 22.84	131.94 ± 24.12	0.020
Total cholesterol, mmol/L	4.26 ± 0.94	4.23 ± 1.13	4.26 ± 1.09	4.84 ± 1.33	0.042
HDL cholesterol, mmol/L	1.05 (0.68–1.99)	1.12 (0.48–2.89)	1.10 (0.07–4.62)	1.02 (0.26–3.4)	0.692
LDL cholesterol, mmol/L	2.45 ± 0.74	2.48 ± 0.90	2.49 ± 0.91	3.27 ± 1.14	<0.001
Triglycerides, mmol/L	1.7 (0.56–5.82)	1.32 (0.54–6.51)	1.46 (0.56–7.93)	1.63 (0.49–6.87)	0.140
ApoA1, mg/dl	1.09 ± 0.17	1.04 ± 0.24	1.08 ± 0.27	1.15 ± 0.24	0.026
ApoB100, mg/dl	0.77 (0.51–1.39)	0.82 (0.33–2.58)	0.88 (0.36–2.58)	1.10 (0.26–2.91)	0.031
Lpa, mg/dl	176 (0–885)	128 (4–1,334)	173 (1.5–1,546)	188 (4–3,440)	0.007
WBC, 10^9^/L	7.33 ± 1.74	7.02 ± 1.72	8.45 ± 2.39	11.94 ± 6.37	<0.001
Hb, g/L	136 (116–169)	136 (69–172)	144 (75–274)	142 (69–274)	0.005
Fasting blood glucose, mmol/L	5.31 (4.15–10.28)	5.36 (4.02–12.79)	5.91 (4.36–15.9)	6.04 (3.7–18.79)	<0.001
Hemoglobin A1c, %	5.85 (5–11)	5.9 (3.46–11.1)	6.1 (4.4–11.1)	6.0 (5.0–14.5)	0.008
Creatinine, mmol/l	71 (43–131)	75 (43–131)	92 (48–187)	81 (42–401)	<0.001
hs-CRP, mg/l	1 (0–67.6)	0.7 (0–12.4)^*^	2.1 (0–54.2)	5.8 (0–183.2)	<0.001
Troponin T, ng/L	40 (3–89)	40 (3–48)	40 (3–2000)	108 (40–3,650)	<0.001
Pro-BNP, pg/ml	60 (24–560)	60 (5–892)	165 (17–9000)	514 (24–11,232)	<0.001
LV diameter, mm	45 (31–59)	45 (28–61)	47 (38–64)	46 (35–69)	0.006
LVEF, %	65 (48–75)	65 (42–79)	62 (30–80)	58 (22–74)	<0.001
Number of lesion vessels	–	1 (1–3)	3 (1–3)	3 (1–3)	<0.001
Antiplatelet therapy	6 (9.6)	86 (93.4)	94 (97)	136 (98)	<0.001
Statins use,% (*n*)	10 (16.1)	94 (98.9)	94 (97)	137 (98.6)	0.187
β blocker use,% (*n*)	14 (22.6)	53 (55.8)	81 (83.5)	115 (82.7)	<0.001
ACEI/ARB use,% (*n*)	10 (15.1)	62 (65.3)	71 (73.2)	97 (69.8)	<0.001
Diuretics use,% (*n*)	6 (9.7)	19 (20)	40 (41.2)	57 (41)	<0.001
Gal-3, ng/ml	2.07 (0.23–9.38)	2.23 (0.65–23.8)^*^	4.47 (0.16–27.1)▴	7.87 (0.59–63.67)※	<0.001

*CAD, coronary artery disease; BMI, body mass index; DM, Diabetic mellitus; WBC, white blood cell; HbA1C, hemoglobin A1C; CRP, C-reactive protein; TC, total chelosterol; HDL-C, high density lipoprotenchlesterol; LDL-C, low density lipoprotein cholesterol; TG, triglyceride; ApoA1, apolipoprotein A1; ApoB, apolipoprotein B; LP(a), Lipoprotein (a); LVEF, left venticular ejection fraction; Gal-3, galectin-3*.

* *means Stable CAD group vs. no CAD group, P = 0.035*.

※ *means STEMI group vs. Stable CAD group, P <0.001*.

▴ *means NSTE-ACS group vs. Stable CAD group, P = 0.04*.

**Table 4:** Logistic regression analysis for risk factors attributing to ACS presence in the study.

	**Univariate analysis**		**Multivariate analysis**	
**Variable**	**OR (95% CI)**	***P*-value**	**OR (95% CI)**	***P*-value**
Gender male sex, % (*n*)	3.784 (2.379–6.018)	<0.001	1.648 (0.599–4.532)	0.333
Smoking, % (*n*)	1.688 (1.109–2.569)	0.015	0.991 (0.455–2.154)	0.981
Systolic blood pressure, mm Hg	0.989 (0.980–0.997)	0.012	0.979 (0.962–0.996)	0.015 ^*^
WBC, 10^9^/L	1.630 (1.448–1.835)	<0.001	1.523 (1.286–1.803)	<0.001^*^
Hb, g/L	1.012 (1.002–1.023)	0.022	1.019 (0.999–1.038)	0.056
Fasting blood glucose, mmol/L	1.338 (1.168–1.532)	0.001	1.313 (1.062–1.623)	0.012 ^*^
Creatinine, μmol/L	1.023 (1.013–1.033)	<0.001	1.017 (0.999–1.035)	0.057
hs-CRP, mg/L	1.082 (1.041–1.124)	<0.001	1.009 (0.976–1.043)	0.602
Totalcholesterol, mmol/L	1.124 (1.047–1.487)	0.013	0.983 (0.515–1.873)	0.957
HDL cholesterol, mmol/L	0.802 (0.487–1.320)	0.386		
LDLcholesterol, mmol/L	1.631 (1.305–2.391)	<0.001	0.959 (0.439–2.096)	0.916
Triglycerides, mmol/L	1.139 (0.944–1.373)	0.174		
ApoA1, mg/dl	3.027 (1.226–7.472)	0.016	4.321 (0.713–26.086)	0.112
ApoB100, mg/dl	7.062 (3.363–14.831)	<0.001	1.907 (0.555–6.549)	0.733
Lpa, mg/dl	1.001 (1.000–1.002)	0.001	1.001 (0.999–1.002)	0.380
LVEF, %	0.000 (0.000–0.004)	<0.001	0.851 (0.804–0.902)	0.006 ^*^
Gal-3, ng/ml	1.141 (1.081–1.204)	<0.001	1.131 (1.051–1.217)	0.001^*^

*CAD, coronary artery disease; BMI, body mass index; DM, Diabetic mellitus; WBC, white blood cell; Hb, hemoglobin; HbA1C, hemoglobin A1C; CRP, C-reactive protein; TC, total chelosterol; HDL-C, high density lipoprotein cholesterol; LDL-C, low density lipoprotein cholesterol; TG, triglyceride; ApoA1, apolipoprotein A1; ApoB, apolipoprotein B; LP(a), Lipoprotein(a); LVEF, leftventicular ejection fraction; Gal-3, galectin-3;*

* *P < 0.05*.

### Serum Galectin-3 Expression in Patients With High vs. Low SYNTAX Scores

**Figure 2:**
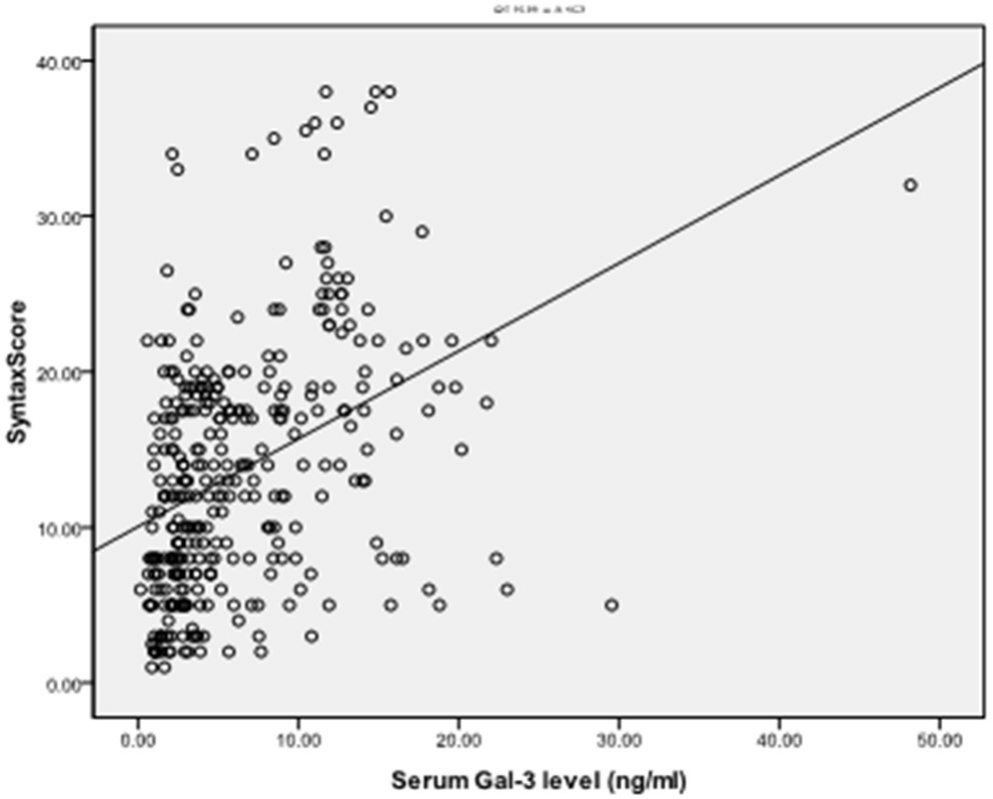
Positive correlation of serum Gal-3 with Syntax Score (SS), *r* = 0.397, *P* < 0.001.

To explore the correlation between the level of serum Gal-3 and the plaque burden of CAD, we calculated the coronary SS of each CAD patient and divided the patients into a low SS (<22, *n* = 248) and a high SS group (≥22, *n* = 83). The serum Gal-3 expression of the high SS group was significantly higher than that of the low SS group 5.62 (1.64–60.15) vs. 3.48 (0.16–63.67) ng/ml, *P* = 0.037, see in Table 5. Spearman correlation analysis showed that the serum Gal-3 levels were positively correlated with SS (*r* = 0.397, *P* < 0.001) (Figure 2). Multivariate logistic regression analysis showed that after adjusting for other risk factors, serum Gal-3 level remained a risk factor for high SS (OR = 1.030, 95% CI: 1.021–1.047, *P* = 0.038) (Table 6).

**Table 5:** The baseline clinical and biochemical characteristics of the low SS group and High SS group.

**Variable**	**SS <22, *n* = 280**	**SS ≥22, *n* = 51**	***P*-value**
Age, years	60.49 ± 11.88	62.49 ± 11.20	0.198
Gender male sex, % (*n*)	222 (79.3)	46 (90.2)	0.048
BMI, kg/m^2^	24.16 ± 2.51	23.81 ± 2.93	0.137
Hypertension, % (*n*)	164 (58.6)	31 (60.7)	0.887
DM, % (*n*)	56 (20)	9 (17.6)	0.848
History of hyperlipidemia, % (*n*)	12 (4.28)	0 (0)	0.225
Smoking, % (*n*)	117 (41.8)	18 (35.3)	0.389
Systolic blood pressure, mm Hg	135.36 ± 22.65	132.19 ± 23.89	0.364
WBC, 109/L	10.03 (4.01–28.7)	11.25 (6.39–21.25)	0.752
Hb, g/L	141 (69–274)	140 (84–223)	0.482
Creatinine, μmol/l	84.72 ± 24.70	90.62 ± 25.70	0.152
hs-CRP, mg/l	2.3 (0–183)	2.25 (0–50)	0.384
Fasting blood glucose, mmol/L	5.8 (3.70–15.24)	6.0 (4.03–18.79)	0.697
Hemoglobin A1c, %	6 (4.6–14.5)	6.0 (4.5–13)	0.325
Total cholesterol, mmol/L	4.52 ± 1.21	4.49 ± 1.35	0.872
HDL cholesterol, mmol/L	1.029 (0.07–4.62)	1.05 (0.17–2.46)	0.159
LDL cholesterol, mmol/L	2.82 ± 1.06	2.80 ± 1.19	0.923
Triglycerides, mmol/L	1.54 (0.49–7.93)	1.38 (0.54–6.87)	0.591
ApoA1, mg/dl	1.07 (0.46–1.99)	1.06 (0.74–1.74)	0.683
ApoB100, mg/dl	0.96 (0.26–2.91)	0.95 (0.41–2.21)	0.864
Lpa, mg/dl	175 (0–3,440)	253 (0–1,961)	0.116
LA diameter, mm	33.28 ± 4.31	33.03 ± 3.06	0.705
LV diameter, mm	46 (35–69)	46 (40–58)	0.592
LVEF,%	57 (31–87)	62 (35–85)	0.595
Gal-3, ng/ml	3.48 (0.16–63.67)	5.62 (1.64–60.15)	0.037

*CAD, coronary artery disease; WBC, white blood cell; Hb, hemoglobin; HbA1C, hemoglobin A1C; CRP, C-reactive protein; TC, total chelosterol; HDL-C, high density lipoprotein cholesterol; LDL-C, low density lipoprotein cholesterol; TG, triglyceride; ApoA1, apolipoprotein A1; ApoB, apolipoprotein B; LP(a), Lipoprotein (a); LVEF, left venticular ejection fraction; Gal-3, galectin-3*.

* *P < 0.05*.

**Table 6:** Univariate and multivaiate logistic regression analysis for risk factors attributing to high SS (SS>22) in the CAD patient.

	**Univariate analysis**		**Multivariateanalysis**	
**Variable**	**OR (95% CI)**	***P***	**OR (95% CI)**	***P***
Gender male sex, % (*n*)	2.146 (1.055–4.367)	0.035	2.418 (1.013–5.773)	0.047^*^
WBC, 10^9^/L	1.071 (1.001–1.146)	0.048	1.040 (0.957–1.130)	0.355
Fasting blood glucose, mmol/L	1.145 (1.031–1.271)	0.011	1.088 (0.951–1.245)	0.220
hs-CRP, mg/l	1.015 (1.001–1.030)	0.039	1.006 (0.991–1.021)	0.462
Total cholesterol, mmol/L	1.071 (0.861–1.333)	0.536		
HDL cholesterol, mmol/L	0.588 (0.271–1.276)	0.179		
LDL cholesterol, mmol/L	1.154 (0.903–1.475)	0.251		
Triglycerides, mmol/L	0.927 (0.723–1.188)	0.548		
ApoA1, mg/dl	0.914 (0.302–2.768)	0.874		
ApoB100, mg/dl	1.641 (0.839–3.207)	0.148		
Lpa, mg/dl	1.00 (1.000–1.001)	0.206		
LV diameter, mm	1.063 (1.006–1.123)	0.030	1.027 (0.955–1.105)	0.470
LVEF,%	0.058 (0.006–0.604)	0.017	0.724 (0.026–20.300)	0.849
Gal-3, ng/ml	1.047 (1.020–1.076)	0.001	1.031 (1.021–1.047)	0.038^*^

*CAD, coronary artery disease; WBC, white blood cell; Hb, hemoglobin; HbA1C, hemoglobin A1C; CRP, C-reactive protein; TC, total chelosterol; HDL-C, high density lipoprotein cholesterol; LDL-C, low density lipoprotein cholesterol; TG, triglyceride; ApoA1, apolipoprotein A1; ApoB, apolipoprotein B; LP(a), lipoprotein(a); LVEF, left venticular ejection fraction; Gal-3, galectin-3*.

* *P < 0.05*.

### The Value of Serum Gal-3 Level in Predicting 1-Year MACEs in ACS Patients

**Figure 3:**
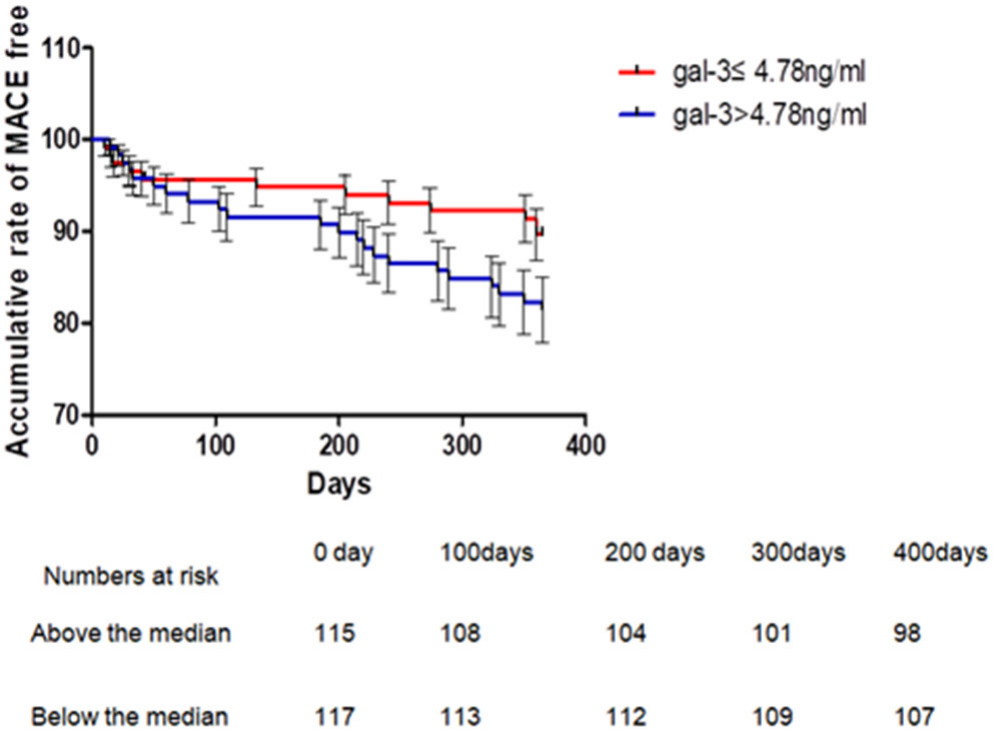
Kaplan-Meier's curves for 1 year MACE according to levels above or below median of serum Gal-3 in patients with ACS. One-year MACE events were significantly higher in Gal-3 >4.78 ng/ml group than that in the Gal-3 = 4.78 ng/ml group, *P* = 0.036 (Breslow). ACS, acute coronary syndrome.

Four patients were lost during follow-up, three of which were in the Gal-3 >4.78 ng/ml group while the other one was in the Gal-3 ≤ 4.78 ng/ml. Kaplan-Meier analysis showed that 1-year MACEs were significantly higher in the high Gal-3 group (Gal-3 >4.78 ng/ml) than in the low Gal-3 group (Gal-3 ≤ 4.78 ng/ml), *P* = 0.036 (Figure 3). Logistic regressions showed that after adjusting other confounding risk factors, Gal-3 remained an independent risk factor for the cumulative rate of MACEs in ACS patients. A 6% higher rate of presence of MACEs per 1 ng/ml increment in Gal-3 level (Table 7).

**Table 7:** Association between serum Gal-3 and incident rate of MACEs.

**Gal-3**	**Incident rate**	**Model 1**		**Model 2**		**Model 3**		**Model 4**	
		**OR (95%CI)**	***P*-value**	**OR (95%CI)**	***P*-value**	**OR (95%CI)**	***P*-value**	**OR (95%CI)**	***P*-value**
Per ng/ml	26/236	1.051 (1.010, 1.094)	0.014	1.065 (1.018, 1.114)	0.007	1.058 (1.011,1.108)	0.015	1.060 (1.010, 1.112)	0.017

*Model 1, adjusted for age, gender; Model 2, further adjusted for Cr, apoB 100; Model 3, further adjusted for fasting glucose, number of lesion vessels; Model 4, further adjusted for N-Terminal Pro-Brain Natriuretic Peptide, Troponin T*.

## Discussion

The main findings of the present study were as follows. (1) Serum Gal-3 was significantly higher in CAD patients than in non-CAD patients and was higher in ACS patients than in stable CAD patients. (2) Galectin-3 was an independent predictor of the presence of CAD as well as the presence of non-stable CAD (ACS). (3) Galectin-3 positively correlated with the complexity of CAD and was an independent risk factor for a high SS. (4) A high level of serum Gal-3 was associated with a higher rate of MACEs in ACS patients over the 1 year of follow up.

Inflammation and oxidative stress play a key role in all stages of atherosclerosis, from initiation to progression of atheromatous plaque, finally leading to ACS (18). In recent years, the role of many new inflammatory-related markers in CVD has been explored, including secret frizzled related proteins (19, 20), gut microbiota produced trimethylamine N-oxide (TMAO) (21), Gal-3 (22), etc. Galectin-3 is a macrophage- and endothelium-derived mediator actively involved in the regulation of many aspects of inflammatory cell behavior (12). It has been found to be involved in proliferation, macrophage chemotaxis, phagocytosis, neutrophil extravasation, and deposition of type-1 collagen in the ECM, resulting in adverse matrix remodeling (23). Clinically, limited data have shown that plasma Gal-3 is significantly higher in CAD patients than in non-CAD patients. However, the precise role of Gal-3 in CAD remains unclear; more data are needed to determine the association between circulating Gal-3 and atherosclerosis. Abayomi Oyenuga et al. showed that higher levels of Gal-3 were associated with greater carotid atherosclerosis (24). In the present study, we found that serum Gal-3 was significantly higher in CAD patients than in non-CAD patients and that Gal-3 was an independent predictor of the presence of CAD. Our data are consistent with previous results (11). Furthermore, we found that Gal-3 levels were positively correlated with WBC count and hs-CRP levels. Although this correlation was weak, these data support the hypothesis that Gal-3 is involved in inflammation and contributes to the formation of atherosclerosis.

Galectin-3 is not only involved in the formation of atherosclerotic plaques but may also contribute to plaque destabilization. To date, it hasn't reached an agreement on the role of Gal-3 on plaque stability, and clinical study and experimental study showed conflicting results (12, 18, 25–29). Current evidence has shown that Gal-3 may play a dual role in plaque instability (22). Our study showed that serum Gal-3 was higher in ACS patients than in stable CAD and non-CAD patients and was an independent predictor of the presence of ACS with a specificity of 79% and a sensitivity of 60% for a cut-off value of 3.93 ng/ml. Our data was in favor of the conception that gal-3 was positively correlated with plaque destabilization. To date, no firm conclusions about the action of Gal-3 (pro-inflammatory vs. anti-inflammatory) during atherosclerosis evolution in rodents have been drawn. More data are needed to clarify the relationship between coronary plaque destabilization and plasma levels of Gal-3.

The SS was developed as a tool to assess the complexity of coronary lesions in the SYNTAX (Synergy between Percutaneous Coronary Intervention with TAXUS and Cardiac Surgery) study. This score adds many characteristics to the simple definition of the number of diseased vessels related to the severity of CAD and has also been indicated to have the prognostic ability (30). To date, few studies have explored the correlation between Gal-3 and SS (18, 31). Aksan et al. found that the plasma concentration of Gal-3 was higher in high SS CAD patients but was not an independent risk factor for high SS after adjusting for other confounding risk factors (19). Turan et al. found that Gal-3 was independently associated with SS (31). In this study, we found that Gal-3 levels were positively correlated with SS. The serum Gal-3 level was shown to be a risk factor for high SS, even after adjusting for other risk factors. The different number of samples and high cut-off SS values may have contributed to the differences in the results. Our data indicated that Gal-3 could be used as a valuable biomarker for the assessment of the severity of CAD.

In our study, we found that serum Gal-3 was positively correlated with LDL-C and ApoB100. It is well known that the interaction between dyslipidemia and inflammation is the basis of atherosclerosis, and many inflammatory factors are involved in the regulation of lipid metabolism disorders (3, 32). This may be evidence that Gal-3 directly regulates cholesterol metabolism, which is an interesting topic that deserves further exploration.

## Limitations

This study has several limitations. First, it is a single center retrospective cohort study and investigated only a relatively small number of patients, further prospective studies with larger sample sizes should be conducted to explore the relationship between Gal-3 and CAD. Second, as it was an observational study, we could not exclude residual confounding factors, despite we had adjusted for potential covariates as much as possible. Thirdly, timing of interventional treatment for ACS was an important confounding risk factor that has an impact on the prognosis. However, data were lacking in our study, which was one of the limitations of the study. Finally, we did not assess serial changes in circulating levels of Gal-3 among STEMI patients and there was evidence that showed that Gal-3 was in dynamic changes during acute phage.

## Conclusion

Galectin-3 correlated with the presence of CAD as well as coronary stability and complexity. Galectin-3 may be valuable in predicting mid-term prognosis in ACS patients.

## Data Availability Statement

The original contributions presented in the study are included in the article/Supplementary Material, further inquiries can be directed to the corresponding author/s.

## Ethics Statement

The studies involving human participants were reviewed and approved by Ethics Committee on Clinical Scientific Research and Laboratory Animal of Zhongshan People's Hospital approved the study. The patients/participants provided their written informed consent to participate in this study.

## Author Contributions

ML wrote and edited the manuscript. KG, XH, LF, YY, JL, and YL collected the research data for the article. ZG reviewed the manuscript and approved the final manuscript. All authors contributed to the article and approved the submitted version.

## Conflict of Interest

The authors declare that the research was conducted in the absence of any commercial or financial relationships that could be construed as a potential conflict of interest.

## Publisher's Note

All claims expressed in this article are solely those of the authors and do not necessarily represent those of their affiliated organizations, or those of the publisher, the editors and the reviewers. Any product that may be evaluated in this article, or claim that may be made by its manufacturer, is not guaranteed or endorsed by the publisher.

## References

[1] Roth GA Johnson C Abajobir A Abd-Allah F Ferede AS Murray C. Global, regional, and national burden of cardiovascular diseases for 10 causes, 1990 to 2015. J Am Coll Cardiol. (2017) 70:1–25. 10.1016/j.jacc.2017.04.052 28527533 PMC5491406

[2] Bonow RO Smaha LA Smith Jr SC Mensah GA Lenfant C. World Heart Day 2002: the international burden of cardiovascular disease: responding to the emerging global epidemic. Circulation. (2002) 106:1602–5. 10.1161/01.CIR.0000035036.22612.2B 12270848

[3] Lusis AJ. Atherosclerosis. Nature. (2000) 407:233–41. 10.1038/35025203 11001066 PMC2826222

[4] Yongsakulchai P Settasatian C Settasatian N. Senthong V. Association of combined genetic variations in PPARγ, PGC-1α, and LXRα with coronary artery disease and severity in Thai population. Atherosclerosis. (2016) 248:140–8. 10.1016/j.atherosclerosis.2016.03.005 27016616

[5] Suthahar N Meijers WC Sillje HH Ho JE Liu FT de Boer RA. Galectin-3 activation and inhibition in heart failure and cardiovascular disease: an update. Theranostics. (2018) 8:593–609. 10.7150/thno.22196 29344292 PMC5771079

[6] Dumic J Dabelic S Flögel M. Galectin-3: an open-ended story. Biochim Biophys Acta. (2006) 1760:616–35. 10.1016/j.bbagen.2005.12.020 16478649

[7] Ruvolo PP. Galectin 3 as a guardian of the tumor microenvironment. Biochim Biophys Acta. (2016) 1863:427–37. 10.1016/j.bbamcr.2015.08.008 26264495

[8] Di Lella S Sundblad V Cerliani JP Guardia CM Estrin DA Vasta GR. When galectins recognize glycans: from biochemistry to physiology and back again. Biochemistry. (2011) 50:7842–57. 10.1021/bi201121m 21848324 PMC3429939

[9] Li M Yuan Y Guo K Huang XS Lao Y Feng L. Value of galectin-3 in acute myocardial infarction. Am J Cardiovasc Drugs. (2019) 20:333–42. 10.1007/s40256-019-00387-9 31784887

[10] Madrigal-Matute J Lindholt JS Fernandez-Garcia CE BenitoMartin A Burillo E Zalba G. Galectin-3, a biomarker linking oxidative stress and infammation with the clinical outcomes of patients with atherothrombosis. J Am Heart Assoc. (2014) 3:e000785. 10.1161/JAHA.114.000785 25095870 PMC4310363

[11] Kusaka H Yamamoto E Hirata Y Fujisue K Tokitsu T Sugamura K. Clinical significance of plasma galectin-3 in patients with coronary artery disease. Int J Cardiol. (2015) 201:532–4. 10.1016/j.ijcard.2015.08.099 26322601

[12] Falcone C Lucibello S Mazzucchelli I Bozzini S D'Angelo A Schirinzi S. Galectin-3 plasma levels and coronary artery disease: a new possible biomarker of acute coronary syndrome. Int J Immunopathol Pharmacol. (2011) 24:905–13. 10.1177/039463201102400409 22230397

[13] Ghorbani A Bhambhani V Christenson RH Meijers WC de Boer RA Levy D. Longitudinal change in galectin-3 and incident cardiovascular outcomes. J Am Coll Cardiol. (2018) 72:3246–54. 10.1016/j.jacc.2018.09.076 30573026 PMC6516745

[14] Ibanez B James S Agewall S Antunes MJ Bucciarelli-Ducci C Buen H. 2017 ESC Guidelines for the management of acute myocardial infarction in patients presenting with ST-segment elevation: The Task Force for the management of acute myocardial infarction in patients presenting with ST-segment elevation of the European Society of Cardiology (ESC). Eur Heart J. (2018). 39:119–77. 10.1093/eurheartj/ehx393 28886621

[15] Roffi M Patrono C Collet JP Mueller C Valgimigli M Andreotti F. 2015 ESC guidelines for the management of acute coronary syndromes in patients presenting without persistent ST-segment elevation: Task Force for the Management of Acute Coronary Syndromes in Patients Presenting without Persistent ST-Segment Elevation of the European Society of Cardiology (ESC). Eur Heart J. (2016). 37:267–315. 10.1093/eurheartj/ehv320 26320110

[16] Task Force Members Montalescot G Sechtem U Achenbach S Andreotti F Arden C. 2013 ESC guidelines on the management of stable coronary artery disease: the Task Force on the management of stable coronary artery disease of the European Society of Cardiology. Eur Heart J. (2013). 34:2949–3003. 10.1093/eurheartj/eht296 23996286

[17] Ludman PF Stephens NG Harcombe A Lowe MD Shapiro LM Schofield PM. Radial versus femoral approach for diagnostic coronary angiography. Am J Cardiol. (1997) 9:1239–41. 10.1016/S0002-9149(97)00089-1 9164893

[18] Tsai TH Sung PH Chang LT Sun CK Yeh KH Chung SY Chua S. Value and level of galectin-3 in acute myocardial infarction patients undergoing primary percutaneous coronary intervention. J Atheroscler Thromb. (2012) 19:1073–82. 10.5551/jat.12856 23037954

[19] Wu J Zheng H Liu X Chen P Zhang Y Luo J. Prognostic value of secreted frizzled-related protein 5 in heart failure patients with and without type 2 diabetes mellitus. Circ Heart Fail. (2020) 13:e007054. 10.1161/CIRCHEARTFAILURE.120.007054 32842761

[20] Huang A Huang Y. Role of Sfrps in cardiovascular disease. Ther Adv Chronic Dis. (2020) 11:2040622320901990. 10.1177/2040622320901990 32064070 PMC6987486

[21] Li W Huang A Zhu H Liu X Huang X Huang Y. Gut microbiota-derived trimethylamine N-oxide is associated with poor prognosis in patients with heart failure. Med J Aust. (2020) 213:374–9. 10.5694/mja2.50781 32959366

[22] Gao Z Liu Z Wang R Yang L. Galectin-3 is a potential mediator for atherosclerosis. J Immunol Res. (2020) 2020:5284728. 10.1155/2020/5284728 32149158 PMC7042544

[23] Aksan G Gedikli Ö Keskin K Nar G Inci S Yildiz SS. Is galectin-3 a biomarker, a player-or both-in the presence of coronary atherosclerosis? J Investig Med. (2016) 64:764–70. 10.1136/jim-2015-000041 26912009

[24] Oyenuga A Folsom AR Fashanu O Aguilar D Ballantyne CM. Plasma galectin-3 and sonographic measures of carotid atherosclerosis in the atherosclerosis risk in communities study. Angiology. (2019) 70:47–55. 10.1177/0003319718780772 29879846 PMC6239970

[25] Menini S Iacobini C Ricci C Blasetti Fantauzzi C Salvi L Pesce CM. The galectin-3/RAGE dyad modulates vascular osteogenesis in atherosclerosis. Cardiovasc Res. (2013) 100:472–80. 10.1093/cvr/cvt206 23975852

[26] Lin YH Lin LY Wu YW Chien KL Lee CM Hsu RB. The relationship between serum galectin-3 and serum markers of cardiac extracellular matrix turnover in heart failure patients. Clin Chim Acta. (2009) 409:96–9. 10.1016/j.cca.2009.09.001 19747906

[27] Papaspyridonos M McNeill E de Bono J Smith A Burnand KG Channon KM. Galectin-3 is an amplifer of infammation in atherosclerotic plaque progression through macrophage activation and monocyte chemoattraction. Arterioscler Thromb Vasc Biol. (2008) 28:433–40. 10.1161/ATVBAHA.107.159160 18096829

[28] Iacobini C Menini S Ricci C Scipioni A Sansoni V Cordone S. Accelerated lipid-induced atherogenesis in galectin-3-deficient mice: role of lipoxidation via receptor-mediated mechanisms. Arterioscler Thromb Vasc Biol. (2009) 29:831–6. 10.1161/ATVBAHA.109.186791 19359660

[29] Kadoglou NP Sfyroeras GS Spathis A Gkekas C Gastounioti A Mantas G. Galectin-3, carotid plaque vulnerability, and potential effects of statin therapy. Eur J Vasc Endovasc Surg. (2015) 49:4–9. 10.1016/j.ejvs.2014.10.009 25457298

[30] Sianos G Morel M-A Kappetein AP Morice M-C Colombo A Dawkins K. The SYNTAX Score: an angiographic tool grading the complexity of coronary artery disease. EuroIntervention. (2005) 1:219–27. 19758907

[31] Turan Y Demir V. The relation of endocan and galectin-3 with ST-segment resolution in patients with ST-segment elevation myocardial infarction. Adv Clin Exp Med. (2020) 29:453–8. 10.17219/acem/118126 32343887

[32] Steinberg D. Atherogenesis in perspective: hypercholesterolemia and inflammation as partners in crime. Nat Med. (2002). 8:1211–7. 10.1038/nm1102-1211 12411947

