# Supplementary material for: Association Between Serum Galectin-3 Levels and Coronary Stenosis Severity in Patients With Coronary Artery Disease
Source: Front Cardiovasc Med. 2022 Feb 7;9:818162. doi: 10.3389/fcvm.2022.818162 (PMC8858949; doi:10.3389/fcvm.2022.818162)
Supplement: Supplementary file 1 [file Table_1.DOCX]

**Supplementary materials of Table 4. The baseline clinical and biochemical characteristics of ACS VS Stable CAD VS non-CAD in the study**

|  | No CAD, n=62 | Stable CAD, n=95 | ACS n=236 | *P value* |
| --- | --- | --- | --- | --- |
| age，years | 60.35±10.36 | 61.00±10.61 | 59.92±19.14 | 0.058 |
| gender male sex，%（n） | 39(62.9) | 57(60) | 211(89.4) | <0.001 |
| BMI， kg/m2 | 24.51±2.22 | 24.18±2.53 | 24.07±2.60 | 0.483 |
| Hypertension，% (n) | 26(41.9) | 59(62.1) | 136(57.6) | 0.026 |
| DM，%（n) | 8(12.9) | 21(21.1) | 44(18.6) | 0.292 |
| History of hyperlipidemia，% (n) | 1(1.6) | 6(6.3) | 6(2.5) | 0.096 |
| Smoking，% (n) | 23(37.1) | 29(30.5) | 106(44.9) | 0.014 |
| Family history of CAD，% (n) | 0(0) | 3(3.1) | 6(2.5) | 0.507 |
| Systolic blood pressure， mm Hg | 142.32±23.33 | 137.93±20.50 | 133.63±23.64 | 0.020 |
| Total cholesterol (mmol/L) | 4.26±0.94 | 4.231±1.13 | 4.60±1.27 | 0.042 |
| HDL cholesterol (mmol/L) | 1.05(0.68-1.99) | 1.12(0.48-2.89) | 1.06(0.07-4.62) | 0.692 |
| LDL cholesterol (mmol/L) | 2.45±0.74 | 2.48±0.90 | 2.95±1.11 | <0.001 |
| Triglycerides (mmol/L) | 1.7(0.56-5.82) | 1.32(0.54-6.51) | 1.58(0.49-7.93) | 0.140 |
| apoA1(mg/dl) | 1.09±0.17 | 1.04±0.24 | 1.12±0.25 | 0.026 |
| apoB100(mg/dl) | 0.77(0.51-1.39) | 0.82(0.33-2.58) | 1.04(0.26-2.91) | 0.031 |
| Lpa(mg/dl) | 176(0-885) | 128(4-1334) | 186(0-3440) | 0.007 |
| WBC， 109/L | 7.33±1.74 | 7.02±1.72 | 10.51±5.40 | <0.001 |
| Hb g/L | 136(116-169) | 136(69-172) | 142(75-274) | 0.005 |
| Fasting blood glucose (mmol/L) | 5.31(4.15-10.28) | 5.36(4.02-12.79) | 6.01(3.7-18.79) | <0.001 |
| Hemoglobin A1c (%) | 5.85(5-11) | 5.9(3.46-11.1) | 6.05(5.06-14.5) | 0.008 |
| Creatinine (mmol/l) | 71(43-131) | 75(43-131) | 83(33-401) | <0.001 |
| hs-CRP (mg/l) | 1(0-67.6) | 0.7(0-12.4) | 4.15(0-183.2) | <0.001 |
| LV Diameter mm | 45(31-59) | 45(28-61) | 46(35-69) | 0.006 |
| LVEF,% | 65(48-75) | 65(42-79) | 60(22-80) | <0.001 |
| Gal-3, ng/ml | 2.07(0.23-9.38) | 2.23(0.65-23.8) | 4.78(0.16~63.67) | 0.001 |

CAD，coronary artery disease；BMI，body mass index；DM，Diabetic mellitus；WBC，white blood cell;HbA1C，hemoglobin A1C;CRP，C-reactive protein;TC，total chelosterol; HDL-C，high density lipoprotenchlesterol； LDL-C，low density lipoprotein cholesterol;TG，triglyceride;ApoA1，apolipoprotein A1; ApoB，apolipoprotein B;LP(a)，Lipoprotein（a）；LVEF，left venticular ejection fraction；Gal-3，galectin-3.
